# The Effects of Probiotic Supplementation on Experimental Acute Pancreatitis: A Systematic Review and Meta-Analysis

**DOI:** 10.1371/journal.pone.0048811

**Published:** 2012-11-13

**Authors:** Carlijn R. Hooijmans, Rob B. M. de Vries, Maroeska M. Rovers, Hein G. Gooszen, Merel Ritskes-Hoitinga

**Affiliations:** 1 Radboud University Nijmegen Medical Centre, SYRCLE at Central Animal Laboratory, Nijmegen, The Netherlands; 2 Radboud University Nijmegen Medical Centre, Department of Epidemiology, Biostatistics and HTA, Radboud University Nijmegen Medical Center, Nijmegen, The Netherlands; 3 Radboud University Nijmegen Medical Centre, Department of Operating Rooms, Radboud University Nijmegen Medical Center, Nijmegen, The Netherlands; University of Valencia, Spain

## Abstract

**Background:**

In February 2008, the results of the PRObiotics in PAncreatitis TRIAl (PROPATRIA) were published. This study investigated the use of probiotics in patients suffering from severe acute pancreatitis. No differences between the groups were found for any of the primary endpoints. However, mortality in the probiotics group was significantly higher than in the placebo group. This result was unexpected in light of the results of the animal studies referred to in the trial protocol. We used the methods of systematic review and meta-analysis to take a closer look at the relation between the animal studies on probiotics and pancreatitis and the PROPATRIA-trial, focussing on indications for harmful effects and efficacy.

**Methods and results:**

Both PubMed and Embase were searched for original articles concerning the effects of probiotics in experimental acute pancreatitis, yielding thirteen studies that met the inclusion criteria. Data on mortality, bacterial translocation and histological damage to the pancreas were extracted, as well as study quality indicators. Meta-analysis of the four animal studies published before PROPATRIA showed that probiotic supplementation did not diminish mortality, reduced the overall histopathological score of the pancreas and reduced bacterial translocation to pancreas and mesenteric lymph nodes. Comparable results were found when all relevant studies published so far were taken into account.

**Conclusions:**

A more thorough analysis of all relevant animal studies carried out before (and after) the publication of the study protocol of the PROPATRIA trial could not have predicted the harmful effects of probiotics found in the PROPATRIA-trial. Moreover, meta-analysis of the preclinical animal studies did show evidence for efficacy. It may be suggested, however, that the most appropriate animal experiments in relation to the design of the human trial have not yet been conducted, which compromises a fair comparison between the results of the animal studies and the PROPATRIA trial.

## Introduction

In February 2008, Besselink et al. published the results of a randomized clinical trial (RCT) on the use of probiotics in patients suffering from severe acute pancreatitis: the PRObiotics in PAncreatitis TRIAl (PROPATRIA) [1]. A total of 296 patients were enrolled in this study, with 152 in the experimental probiotic group and 144 in the placebo group. The study product, a food supplement called Ecologic 641 (10^10^ bacteria) or placebo was administered twice daily and added to the continuously running fibre-enriched tube feeding for a maximum of 28 days. No differences between the groups were found for any of the primary endpoints (infected pancreatic necrosis, bacteraemia, pneumonia, urosepsis or infected ascites). Pathogens cultured from the 87 patients with an infectious complication showed no significant differences between the groups. However, mortality in the probiotics group was significantly higher than in the placebo group (16% vs. 6%, respectively).

This result was unexpected in light of the results of the animal studies referred to in the trial protocol (the English protocol [2] cites Mangiante et al. 2001 [3], the Dutch protocol [4] refers to Mangiante et al. 2001 [5] and Lutgendorff et al. 2006 [6]). Lack of correspondence between animal data and results from clinical trials is not uncommon. It has been suggested that in order to increase the potential value of animal studies as a preparation for clinical applications not only the methodological quality of the individual animal studies needs to be improved [7], [8], [9], but systematic reviews (SRs) should become the standard method for analysing preclinical studies in relation to one another. By means of such reviews (particularly if they include a meta-analysis), information relevant for judging the safety and efficacy of drugs/treatments may be obtained that is not directly visible from the individual animal studies [10], [11].

Therefore, in this paper, we used the methods of systematic review and meta-analysis to take a closer look at the relation between the animal studies on probiotics and pancreatitis and the PROPATRIA-trial. We focussed on two questions: (1) Could a more thorough analysis of the animal studies carried out before the start of the trial have revealed indications for the harmful effects of probiotics found in the PROPATRIA-trial? and (2) What would the result of such an analysis be regarding the overall efficacy of probiotics on the main outcome measures of the PROPATRIA-trial (mortality, histopathology of the pancreas, bacterial translocation to the pancreas or the mesenteric lymph nodes)? Moreover, given that after the start and the completion of the PROPATRIA-trial, more animal experiments on the subject were published, we tried to answer the same questions taking all animal experiments on probiotics and pancreatitis into account.

## Materials and Methods

### 1. Search Strategy and Selection of the Papers

We searched PubMed and Embase for original articles concerning *the effects of probiotics on experimental acute pancreatitis* until August 5, 2011. The search strategy was composed of three elements: pancreatitis, probiotics, and animals (for complete search strategy see Table 1). It was developed in cooperation with experts/information specialists from the Medical Library of the Radboud University Nijmegen, the Netherlands. To detect all animal studies in both PubMed and Embase, search filters were used [12], [13]. Furthermore, the reference lists of the selected relevant papers were screened by hand for potentially relevant new papers. No language restriction was used. If necessary, papers in languages other than English were translated by scientists (native speakers for that particular language) within the Radboud University Nijmegen Medical Centre. The selection of studies was performed on the basis of the title and abstract. In case of doubt, the entire publication was purchased and evaluated. Two investigators (C. Hooijmans and R. de Vries) independently screened all the abstracts for the inclusion criteria. Differences were resolved by a third investigator (M. Ritskes-Hoitinga). Studies were included if they studied the effects of probiotics on mortality, histopathology of the pancreas or bacterial translocation to the pancreas or mesenteric lymph nodes(MLN), in experimental acute pancreatitis. Papers were excluded if they fulfilled one of the following criteria: (1) Not an original paper (e.g. review or letter etc.); (2) Probiotic supplementation was combined with other (nutritional) components; (3) Double publication; in case a paper occurred more than one time in one of the databases, only the original manuscript was included. The inclusion criteria and methods of analysis were specified in advance and documented in a protocol.

**Table 1:** Search strategy.

PubMed	
Component 1: pancreatitis	"pancreatitis"[MeSH Terms] OR "pancreatitis"[tiab] OR "ANP"[tiab] OR "Pancreatitides"[tiab] OR ("pancreas"[tiab] AND "inflammation"[tiab])
Component 2: probiotics	"probiotics"[MeSH Terms] OR "probiotics"[tiab] OR "probiotic"[tiab] OR "bifidobacterium"[MeSH Terms] OR "bifidobacterium"[tiab] OR "bifidobacteria"[tiab] OR "lactobacillus"[MeSH Terms] OR "lactobacillus"[tiab] OR "saccharomyces"[MeSH Terms] OR "saccharomyces"[tiab] OR "sporobacterin"[Substance Name] OR "sporobacterin"[tiab] OR "bacillus subtilis"[MeSH Terms] OR ("bacillus"[tiab] AND "subtilis"[tiab]) OR "lactococcus lactis"[MeSH Terms] OR ("lactococcus"[tiab] AND "lactis"[tiab]) OR "synbiotic"[tiab] OR "synbiotics"[tiab] OR "lactic acid bacteria"[tiab]
Component 3: animal	Search filter for animal studies [13]
Embase	
Component 1: pancreatitis	(exp pancreatitis/OR pancreatitis.ti,ab. OR pancreatitides.ti,ab. OR ANP.ti,ab. OR (pancreas.ti,ab. AND inflammation.ti,ab.) OR (pancreatic.ti,ab. AND inflammation.ti,ab.))
Component 2: probiotics	(exp probiotic agent/OR probiotics.ti,ab. OR probiotic.ti,ab. OR probiotica.ti,ab. OR exp synbiotic agent/OR synbiotic.ti,ab. OR synbiotics.ti,ab. OR exp bifidobacterium/OR bifidobacterium.ti,ab. OR bifidobacteria.ti,ab. OR exp lactobacillus/OR lactobacillus.ti,ab. OR lactobacilli.ti,ab. OR lactobacterium.ti,ab. OR lactobacteria.ti,ab. OR exp lactococcus/OR lactococcus.ti,ab. OR lactococci.ti,ab. OR exp bacillus/OR bacillus.ti,ab. OR bacilli.ti,ab. OR exp saccharomyces/OR saccharomyces.ti,ab. OR sporobacterin.ti,ab. OR exp lactic acid bacterium/OR lactic acid bacteria.ti,ab. OR lactic acid bacterium.ti,ab. OR Nissle 1917.ti,ab.)
Component 3: animal	Search filter for animal studies [12]

### 2. Study Characteristics and Data Extraction

From the studies included, the following data were extracted: animal species, strain, age or body weight of animals at the beginning of the study, gender, description of control group, method of AP induction, type and dose of probiotics, timing of probiotic supplementation relative to AP induction, duration of probiotic supplementation, route of administration, timing of data collection, number of animals in treatment and control group, number of animals excluded for statistical analysis, reason for excluding animals, outcome measures (Table 2). Bibliographic details such as author, journal, year of publication and original language were also registered. Four outcome measures were included in the meta-analysis: mortality, bacterial translocation to the pancreas and MLN and histopathology of the pancreas. In order to assess the pathology of the pancreas overall pathology scores were recorded. In case only specific pathology scores were presented (e.g. inflammation and parenchymal necrosis) an overall score with its variance was calculated by averaging all separate means by uniform weighing [14]. For all studies, number of events or mean, standard deviation (SD) or standard error (SE) and total number of animals per group were recorded. If data were only presented graphically, attempts were made to obtain data from the authors; if these data were not made available, data were measured using an universal on-screen digitizer where possible (Universal Desktop Ruler). With this software it is possible to measure distances, areas and perimeters of figures on a computer screen.

**Table 2:** Study characteristics of the included studies.

Reference	Language	Species/strain	Sex	Control group	n(c)/n(exp)	Method AP induction	Type of prob.	Timing prob suppl.	Duration prob. suppl.	Dose (per day)	Admin. route	Timing data coll.	Outcome measures
Akyol 2003	English	Rat/SD	M	AP+ placebo	20/20	3% sodium taurocholate(id)	S.boulardii	6 h after AP	42 hours	25 mg	oral	48 h after AP	HP pancreas BT pancreas Mortality
Chen 2007 (#)	Chinese	Rat/SD	M	AP (∞)	8/8	3.8% sodium taurocholate beneath pancreatic capsule	L.lactisL.acidophilus S.lactis	*	4 days or 7 days/?	2 tablets/day (10^9^ CFU/tab.)	*	4 and 7 days after AP	BT MLN
Deng 2000	Chinese	Dog/hybrid	*	AP	8/8	Retrograde perfusion of artificial bile into pancreatic duct and 5% sodium taurocholate + trypsin (3000 U/kg) (id)	Double forked bacilli L.acidophilus enterococci	after AP	7 days/?	0.5gr/kg	oral	1,2,4 and 7 days after AP	BT pancreas +MLN
Horst 2009	English	Rat/Wistar	M	AP	10/10	5% sodium taurocholate(id)	L.rhamnosus L.casei L.acidophilus B.longum	14 days before AP	14 days	1.2×10^9^ CFU	ig	12 h after AP	BT pancreas +MLN HP pancreas
Karen 2010	English	Rat/Wistar	M	AP+ placebo	10/10	glycodeoxycholate (id) (1.2 ml/kg) and 5 µg/kg/h cerulein (iv)	S.boulardii	6 h and 24 h after AP	2 days	25 mg/kg	gavage	2 days after AP	BT pancreas +MLN HP pancreas
Lutgendorff 2008	English	Rat/SD	M	AP+ placebo	12/12	glycodeoxycholate(id) (15 mM) and 5 µg/kg/h cerulein (iv)	L.acidophilus L.casei L.salivarius L.lactis B.bifidum B.lactis	5 days before AP	5 days before until 6 h after	5.0×10^9^ CFU	ig	2 days after AP	HP pancreas
Mangiante 2001	English	Rat/Lewis	*	AP	20/20	Ligation of the biliopancreatic duct	L.plantarum	4 days before until 4 days after AP	8 days	0.5×10^9^ CFU	gavage	4 days after AP	BT pancreas + MLN
Muftuoglu 2006	English	Rat/Wistar	*	AP	10/10	(i.p) injection of arginine (250 mg/100g)	S.thermophilus L.acidophilus B.lactis	*/after	5 days	200 mg 2.4×10^9^ CFU	oral gastric tube	5 days after AP	HP pancreas BT pancreas
Qin 2006	Chinese	Rat/SD	M	AP+ placebo	7/8	3% sodium taurocholate(id)	L.plantarum	6 days afterAP	6 days	1.0×10^7^ CFU	Iv and je-jenum tubes	6 days after AP	BT MLN Mortality
Sahin 2007	English	Rat/Wistar	F	AP+ placebo	10/10	glycodeoxycholate (id) (1.2 ml/kg) and 5 µg/kg/h cerulein (iv)	S.boulardii	6 h and 30 hours after AP	6 or 30 hours	3 ml 25 mg/kg	oral	2 days after AP	HP pancreas
Tarasenko2000(#)	English	Rat/August	M	AP (¤)	3?/3?	bile with a drop of autological blood (id) and mechanical damage to pancreas	B.subtulis	before AP	*	*	*/gastric tube?	2, 24 and 48 h after AP	BT pancreas +MLN
Van Minnen 2006	English	Rat/SD	M	AP+ placebo	21/17(11/13) Δ	glycodeoxycholate (id) (0.5 ml; 10 mmol/l) and cerulein (iv) (5 ug/kg/h for 6 h)	L.acidophilus L.casei L.salivarius L.lactis B.bifidum B.infantis	5 days before AP until 7 days after AP	2 weeks	1×10^10^ CFU	ig	7 days after AP	BT pancreas +MLN Mortality
Yang 2006	Chinese	Rat/SD	M	AP	?/?	5% sodium taurocholate(id)	L.plantarum	2 days after AP	5 days	10 ml 10^6^ CFU/ml	Cathe-ter jejunos tomy	7 days after AP	Mortality BT MLN

? = unclear, * = not mentioned, # = study consists out of multiple experiments (Chen et al;4 exp., Tarasenko et al. 3 exp), Δ = in statisitcal anlysis different numbers of animals used, ∞ = study contains 4 experiments, and thus also 4 control groups: AP parenteral nutrition for 4 days, AP parenteral nutrition for 7 days : AP enteral nutrition for 4 days, AP enteral nutrition for 7 days, (¤) all animals receive labeled e-coli as well, SD = spraque dawley rat, M = male, F = female, id. = intraductally, iv = intravenously, AP = acute pancreatitis, MLN = mesenteric lymph nodes, HP = histpathology, BT = bacterial translocation.

### 3. Assessment of Risk of Bias in Included Studies

We assessed the risk of bias of the included studies using the criteria/items described in Table S1. We based these criteria on the possible presence of selection bias (items 1, 2 and 3), performance bias (items 4 and 7), detection bias (items 5, 6 and 8) and attrition bias (items 9 and 10) [15]. The criteria were independently assessed by two reviewers (C. Hooijmans and R. de Vries) by using collectively predefined judging criteria. The score “yes” indicates low risk of bias, the score “no” indicates high risk of bias, “?” indicates unknown risk of bias.

### 4. Data Synthesis and Statistical Analysis

For the outcome measure ”histopathology of the pancreas”, the standardized mean difference (SMD) was calculated (the mean of the experimental group minus the mean of the control group divided by the pooled SD of the two groups), for all other outcome measures (i.e. bacterial translocation and mortality) the Odds Ratio was determined. If continuous data were presented, data were discussed and presented in the tables but not included in meta-analysis. Where outcomes were measured repeatedly on different time points, we used the time point at which the measured efficacy was greatest [16]. In one study, histopathological data was presented as median and percentiles, these data were converted to mean and SD [17]. In case histopathological data was not presented in an overall score, we calculated an overall score by uniformly weighing the separate means and SE’s of fibrosis, acinar cell loss etc.

Despite anticipated heterogeneity, the individual effect sizes (either OR or SMD) were pooled whenever possible (starting from two studies or more) to obtain an overall effect size and 95% confidence interval. We used the random effects model [18], which accounts for anticipated heterogeneity. Subgroup analyses were planned for the following study characteristics: year of publication (before or after the publication of the trial protocol of Besselink et al in 2004 [2]) and study design (comparable design to Besselink et al [2]). In order to explore possible causes for heterogeneity, subgroup analyses were planned for the following study characteristics: timing of probiotic supplementation (before or after induction of AP), type of probiotic supplement (multi strains vs single strain).

The subgroup analyses were only performed if the overall meta-analysis contained a minimum of 4 studies. Since there are relatively few studies in each subgroup, the estimates of the variances within a subgroup are not likely to be reliable. In addition, we expected the variance to be comparable within the 2 subgroups; therefore, we assumed a common among study variance across subgroups. Because of low power, no statistical tests were used to confirm differences between subgroups.

To detect publication bias, funnelplots were created and explored. Meta-analysis was performed using Comprehensive Meta Analysis (CMA version2.0). Forest plots were used to display the mean overall effect sizes, together with effect sizes for subgroups.

In order to assess the robustness of our findings and in an attempt to further explain observed study heterogeneity, we performed a sensitivity analysis and we investigated the effect of possible interactions by species and quality. Because of the small number of experiments in these subgroups interactions, the results of this sensitivity analysis and interactions should be interpreted with caution.

## Results

### 1. Description of the Included Studies

**Figure 1:**
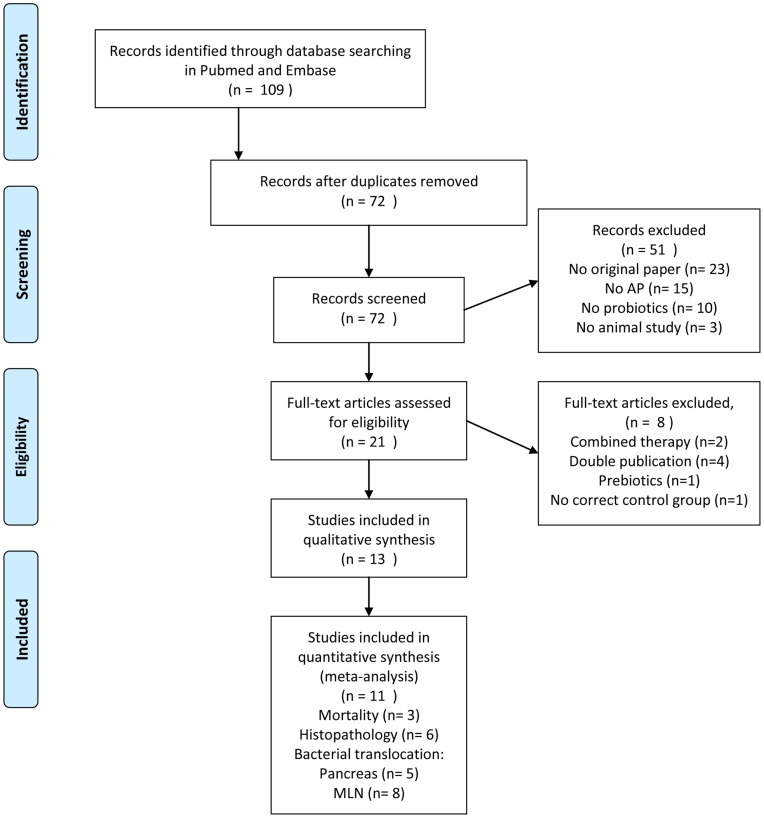
Flow diagram of the systematic review and meta-analysis literature search results.

The search strategy described in Table 1 retrieved 38 papers in PubMed and 71 papers in Embase. Initially, 21 papers seemed to meet our selection criteria. After studying the full-text articles, 13 original studies remained [5], [14], [16], [17], [19], [20], [21], [22], [23], [24], [25], [26], [27] (Fig. 1).

The characteristics of these studies are shown in Table 2. Four of these studies needed to be translated since they were published in Chinese [21], [22], [24], [27]. The study characteristics varied considerably between the included papers. Twelve studies were performed with rats and one used dogs. Nine studies used only males, one study used females, and three papers did not mention the gender of the animals. Seven different techniques were used to induce AP. Also the timing of probiotic supplementation varied greatly between the studies. Six papers mentioned supplementing probiotics after AP induction, 4 studies before AP induction and 3 studies started probiotics supplementation before AP induction and continued supplementation until a few days after AP induction. In four experiments the effects of probiotic supplementation on mortality in experimental acute pancreatitis were studied. Seven studies presented an overall histopathological score of the pancreas, of which six could be included in the meta-analysis. Eleven experiments (extracted from 8 papers) studied the effects of probiotics on bacterial translocation (BT) to the MLN. Eight of the experiments could be included in the meta-analysis. Eight studies studied the effect of probiotics on BT to the pancreas, five of these studies could be included in the meta-analysis.

### 2. Risk of Bias and Quality of Reporting

**Figure 2:**
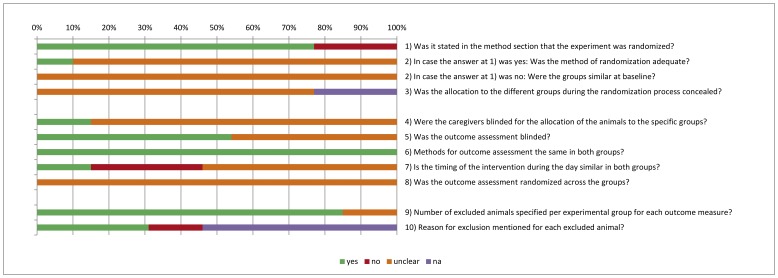
Risk of bias, averaged per item. yes = low risk of bias, no = high risk of bias, ? = unclear risk of bias, n.a. = not applicable.

Figure 2 shows the overall results of the risk of bias assessment of the 13 studies included in this SR. 77% of the studies stated that the allocation of the experimental units to the treatment groups was randomized. However, only two of these studies mentioned the method of randomization used and only one provided sufficient details so that the adequacy of the method could be judged. None of the papers described whether or not the allocation to the different groups during the randomization process was concealed. 54% of the studies reported that they blinded the outcome assessment. Table S1 shows that only four out of the 13 studies scored 5 out of the 10 items as low risk of bias. All of these papers were written in the English language, and none of them were published before the study protocol of Besselink *et al*. In addition, Figure 2 clearly shows that many items were scored as “unclear risk of bias”, which indicates poor reporting of animal studies in scientific publications. This is also highlighted in Table 2, which shows, for example, that in 31% of the studies the exact timing of probiotic supplementation relative to AP induction was not clear and in 23% of the studies the gender of the included animals was not mentioned.

### 3. Publication Bias

The presence of publication bias was assessed for the outcome measures BT MLN and BT liver since those outcomes contained at least ten or more studies. However, the variation in SE was too small to interpret the funnel plots reliably (data not shown).

### 4. Effects of Probiotic Supplementation

#### 4.1. Mortality

**Figure 3:**
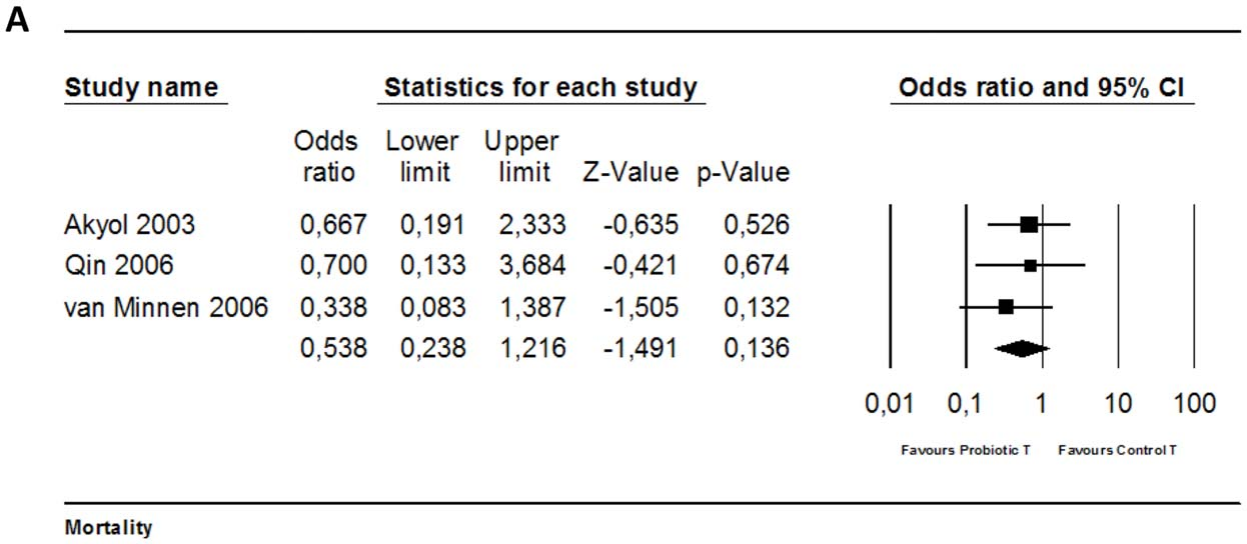
Effects of probiotic supplementation on mortality in experimental acute pancreatitis. Forest plot of the data of three included studies. The forest plot displays the OR, 95% confidence interval and relative weight of the individual studies. The diamond indicates the global estimate and its 95% confidence interval.

Four experiments studied the effect of probiotic supplementation on mortality in experimental AP. Three of these studies could be included in the meta-analysis (as in one study the number of animals per group was unclear). None of these three studies showed a significantly reduced risk on mortality due to probiotic supplementation in experimental AP. Meta- analysis also showed no effect (Fig. 3; OR 0.54 [0.24, 1.22]; n = 3). Heterogeneity was low (Q = 0.62, p = 0.73; I^2^ = 0.0%), and all included studies used rats.

The study of Yang et al 2006 [27], which could not be included in meta-analysis, also showed no significant reduction in mortality due to probiotic supplementation in experimental AP.

#### 4.2. Histopathology of the pancreas

**Figure 4:**
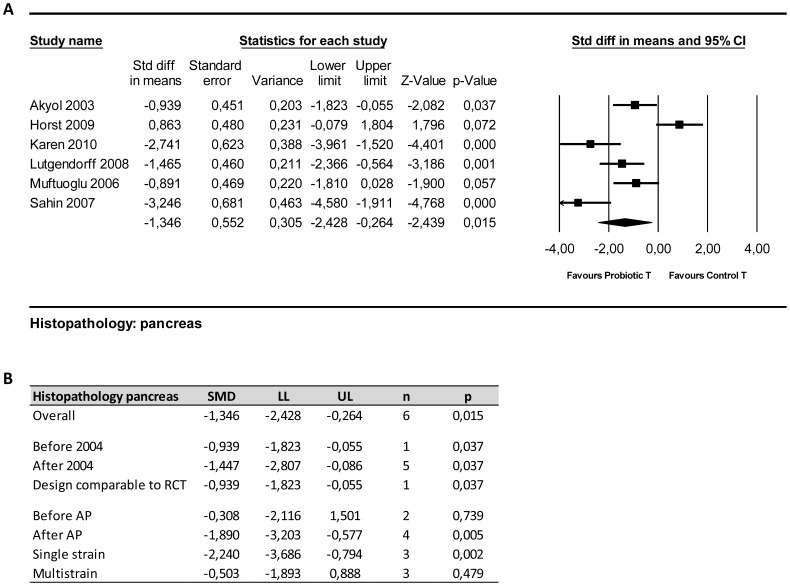
Effects of probiotic supplementation on histopathological damage to the pancreas in experimental acute pancreatitis. (a) Forest plot and (b) subgroup analysis of the data of six included studies. The forest plot displays the SMD, 95% confidence interval and relative weight of the individual studies. The diamond indicates the global estimate and its 95% confidence interval.

Six out of 7 papers investigating the effect of probiotic supplementation in experimental AP on histopathological abnormalities in the pancreas could be included in meta-analysis. Four of these papers showed a significant reduction of the total histopathological score due to probiotic supplementation compared to controls. Overall analysis also showed that probiotic supplementation reduced/improved the overall histopathological score of the pancreas (Fig. 4; SMD −1.35 [−2.43, −0.26]; n = 6; p = 0.015). Heterogeneity was high (Q = 34.53, p<0.01; I^2^ = 85%), although all studies were performed in one species (rats).

Before the publication of the study protocol, solely the study of Akyol [20] had been published (Fig. 4b). This specific paper showed lower histopathological scores in the probiotic group as compared to controls (p = 0.037). The results of the study of Akyol were in accordance with the results of the overall analysis [20].

Subgroup analysis also revealed that in studies supplementing probiotics after AP induction a significant decrease in the histopathological score was present, whereas in studies supplementing probiotics before AP no significant decrease was observed (Fig. 4; after; SMD −1.89 [−3.02, −0.58]; n = 4′; p<0.01before; SMD −0.31 [−2.11, 1.50]; n = 2; p = 0.74).

Subgroup analyses on the study characteristic “type of probiotic supplement” showed that single strain supplementation reduced the overall histopathological score of the pancreas, whereas multistrain supplementation did not (Fig. 4b). Subgroup analysis did not reduce heterogeneity.

#### 4.3. Bacterial translocation to the pancreas

The effect of probiotic supplementation on bacterial translocation (BT) to the pancreas was studied in eight papers. Five of these studies presented their data as a binary outcome (presence or absence of bacterial translocation) and three others presented their data as a continuous variable (amount of colony forming units/g). Only binary outcomes were included in the meta-analysis.

**Figure 5:**
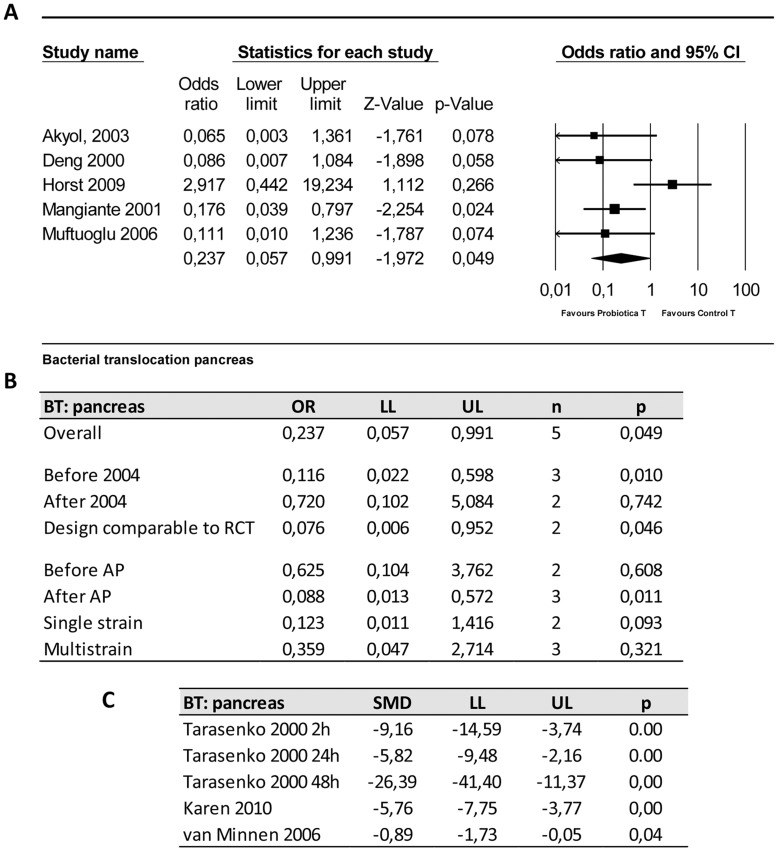
Effects of probiotic supplementation on bacterial translocation to the pancreas in experimental acute pancreatitis. (a) Forest plot and (b) subgroup analysis of the data of five included studies. The forest plot displays the OR, 95% confidence interval and relative weight of the individual studies. The diamond indicates the global estimate and its 95% confidence interval. (c) effectsizes and 95% confidence interval of three studies presenting continuous data which were not included in meta-analysis in order to reduce heterogeneity.

The five experiments that could be included in the meta-analysis showed that the odds of BT to the pancreas is less likely to occur in the probiotic group as compared to the control treated groups (OR 0.24 [0.06, 0.99]; n = 5; p = 0.049). Heterogeneity was moderate (Q = 8.57, p = 0.073; I^2^ = 53%). Three studies using continuous variables for BT to the pancreas also showed significant reductions in the number of bacteria translocated to the pancreas in the probiotic group (Fig. 5c).

Before the publication of the trial protocol by Besselink et al 2004 [2], four studies concerning probiotic supplementation in experimental AP were published. Meta-analysis, which could include 3 of these studies, revealed that at that time the overall effect on BT to the pancreas was already significant (OR 0.12 [0.02, 0.60]; n = 3; p = 0.01). Two of these studies were really indicative for the planned trial, because those studies supplemented probiotics after induction of AP. Subgroup analysis of these 2 studies showed that there was at that time already a significantly reduced risk of BT to the pancreas in probiotic treated animals with AP (OR 0.08 [0.01, 0.95]; n = 2; p = 0.046).

Comparison of the effects of probiotic supplementation before or after inducing experimental AP on the risk of BT to the pancreas revealed that supplementation of probiotics before inducing AP has no significant effect (OR 0.63 [0.10, 3.76]; n = 2; p = 0.61) in contrast to supplementation of probiotics after inducing AP (OR 0.09 [0.03, 0.57]; n = 3; p = 0.01).

Subgroup analysis on the timing of probiotic supplementation did not reduce heterogeneity substantially, and remained moderate. Subgroup analyses on the study characteristic “type of probiotic supplement” showed also here that single strain supplementation might reduce the risk of BT to the pancreas in probiotic treated animals with AP, whereas with multistrain supplementation no such an effect could be observed (Fig. 5b). Heterogeneity levels clearly decreased in the subgroup single strain (Q = 0.33, p = 0.56; I^2^ = 0.0%).

**Figure 6:**
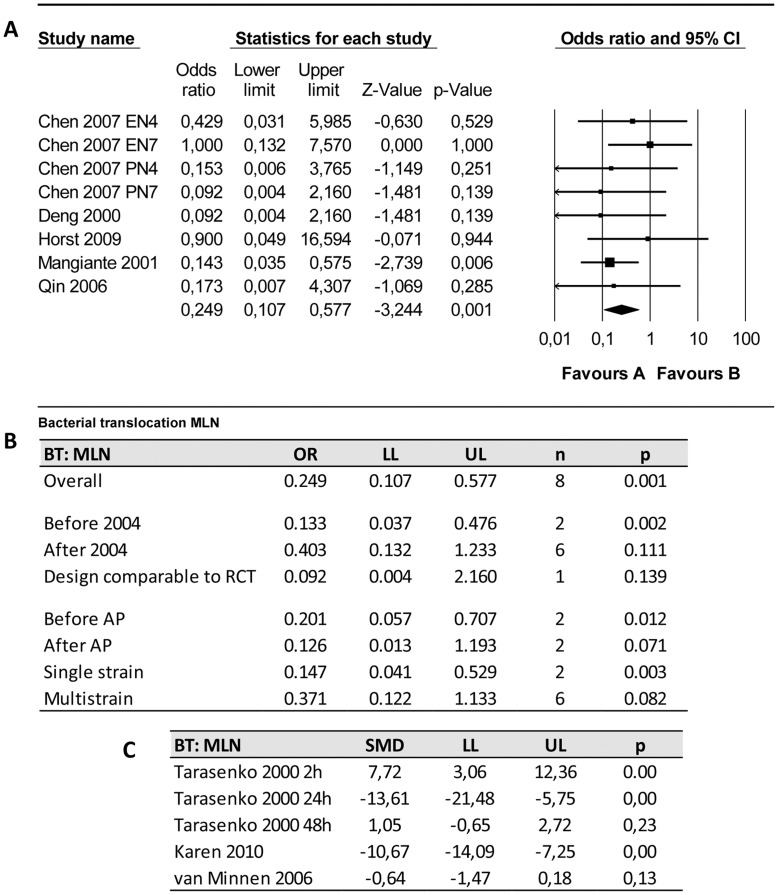
Effects of probiotic supplementation on bacterial translocation to the mesenteric lymph nodes (MLN) in experimental acute pancreatitis. (a) Forest plot and (b) subgroup analysis of the data of eight included experiments. The forest plot displays the OR, 95% confidence interval and relative weight of the individual studies. The diamond indicates the global estimate and its 95% confidence interval. (c) effectsizes and 95% confidence interval of three studies presenting continuous data which were not included in meta-analysis in order to reduce heterogeneity.

#### 4.4. Bacterial translocation to the mesenteric lymph nodes (MLN)

The effect of probiotic supplementation on bacterial translocation (BT) to the MLN was studied in nine papers. Five of these studies (including 8 experiments) presented their data as a binary outcome and three others presented their data as a continuous variable (amount of colony forming units/g). Only binary outcomes were included in the meta-analysis.

Overall analysis revealed that probiotics reduced the risk of BT to the MLN (Fig. 6a; OR 0.25 [0.11, 0.58]; n = 8; p<0.01). Heterogeneity was low (Q = 4.23, p = 0.753; I^2^ = 0.0%).

Before the publication of the study protocol of Besselink et al. [2] three studies investigated this outcome measure as well (of which 2 could be included in meta-analysis). However, only one study had a comparable design (i.e. probiotic supplementation after AP induction). This study showed no significant reduction of the risk of BT to the MLN due to probiotic supplementation (OR 0.09 [0.01, 2.16]; p = 0.14).

Subgroup analysis (Fig. 6b) on the effects of the timing of probiotic supplementation relative to AP induction could only be performed with 4 studies, because the other 4 experiments did not describe the timing of supplementation. This analysis showed that both probiotic supplementation before and after AP induction could be beneficial (Fig. 6b ). The results of the studies presenting continuous data (except for van Minnen et al [26] and Tarasenko et al [16], after 2 h) are in accordance with the results of the meta-analysis (Fig. 6c).

Subgroup analyses on the study characteristic “type of probiotic supplement” showed that both single strain supplementation and multistrain supplementation seem to reduce BT to the MLN (Fig. 6b). The effect appears to be larger in the single strain group as compared to the multistrain group (single; OR 0.15 [0.05, 0.53]; n = 2; p<0.01, multi: 0.371 [0.12, 1.11]; n = 6; p = 0.08).

### 5. Sensitivity Analysis and Interactions

To assess the robustness of our findings and in an attempt to further explain the observed study heterogeneity, we performed a sensitivity analysis and we investigated the effect of possible interactions by species and quality.

In the analysis of outcome measures BT to the pancreas and MLN also a dog study was included. Analyzing the outcome measures without this dog study resulted in different results for the outcome measure BT to pancreas. The odds of BT to the pancreas was no longer less likely to occur in the probiotic group as compared to the control groups (OR 0.29 [0.05, 1.57]; n = 4; p = 0.15). Heterogeneity remained moderate (Q = 7.67, p = 0.05; I^2^ = 60%).

Sensitivity analysis revealed that excluding the studies with an overall quality score lower than 60% (Table 2) solely altered the results of BT to the pancreas. The excluded study for this outcome measure was again the dog study and therefore the sensitivity analysis showed the same results as mentioned above.

## Discussion

The results of this SR and meta analysis show that a more thorough analysis of all relevant animal studies carried out before the start of the PROPATRIA trial [1], would not have revealed indications for harmful effects of probiotics. A combination of the results of all four animal studies published before the trial did not show an adverse effect on the main outcome measure (i.e. mortality). Moreover, it did show a positive effect on other outcome measures, namely improved histopathology of the pancreas and reduced bacterial translocation to the pancreas and to the MLNs. These overall conclusions do not change when all animal studies on probiotics and pancreatitis published so far – including the ones performed after the PROPATRIA trial - are taken into account.

The PROPATRIA-trial in humans showed higher mortality in the group treated with probiotics [1]. This result was unexpected in light of the results of the animal studies referred to in the trial protocol by Besselink et al. [2], [4]. The protocol referred (indirectly) to one animal study published before the start of the trial (namely Mangiante et al. 2001 [5]), which showed some evidence for reduced bacterial translocation to the pancreas and MLN, but did not study the effect of probiotics during AP on mortality. We were able to identify three other animal studies that had already been published at the time (Deng et al. 2000 [22], Tarasenko et al. 2000 [16], Akyol et al. 2003 [20]). Of these three, only one study (Akyol et al. 2003 [20]) reported data on mortality and this study showed a non-significant reduction of mortality. Because mortality was not intended to be a primary outcome measure in most of the animal studies, the experiments may have been underpowered to be able to detect a possible significant difference in mortality between probiotic treated and control groups. Even though Tarasenko et al. 2000 [16] and Mangiante et al. 2001 [5] did not provide data on mortality, the former emphasized that *B. subtilis* did not aggravate the course of experimental AP and the latter stressed that, as a proof of the safety of the probiotic prophylaxis, they never found *L. plantarum* in blood samples, also not in samples from animals with BT. In other words, although only very few animal experiments had been carried out and data on mortality available were not fully reliable, there were at the start of the PROPATRIA trial no indications from animal experiments that probiotics might have a harmful effect. None of these four studies, however, supplemented the probiotic Ecologic 641, which was used in the trial.

Also inclusion of more recent animal studies does not provide evidence for an increase in mortality due to probiotic supplementation. Nevertheless, we found one study that reported a tendency towards adverse effects of probiotics: Horst et al. (2009) [23] showed a higher histopathological score and an increase in bacterial translocation to the pancreas after probiotic supplementation. Both effects were not statistically significant, however, and did not alter the direction of the overall effect in the meta-analysis. The most striking differences between the study of Horst et al. [23] and the other studies are the duration of probiotic supplementation before AP induction (14 days in the Horst study; on average 4–5 days in the other studies) and timing of the outcome assessment (12 hours after AP induction in Horst et al.; on average 4 days after AP induction in all other studies). However, although the results of the study of Horst et al. were closer to the clinical truth, the above mentioned differences do not offer an obvious explanation for this.

With regard to the efficacy of probiotics in AP, the combined analysis of the four animal studies executed before the start of the trial showed that probiotic supplementation did not affect mortality, but led to an improved histopathological score for the pancreas and reduced bacterial translocation to pancreas and MLNs. These results remained largely the same when only the studies were taken into account that used probiotic supplementation after induction of AP (so with a design comparable to the PROPATRIA-trial). In addition, including also the more recent studies does not alter these results. In summary, the animal studies on probiotics and AP showed improvement in two important clinically relevant outcome measures, namely pancreatic histopathology and reduced bacterial translocation, and had no effect on the main outcome measure, mortality.

Most of the studies were originally not designed to measure an effect of probiotics on mortality and might for this reason be underpowered. Moreover, the timing of the determination of mortality should be taken into account. In humans, acute pancreatitis typically follows a biphasic course: the early phase is associated with systemic inflammatory response syndrome (SIRS), (multiple) organ damage and early mortality (<1 week), the late phase is characterized by infectious complications following bacterial translocation of intestinal bacteria and late mortality (>3 weeks). Given that only the late mortality is mediated by infection/bacterial translocation, an effect of probiotics on early mortality is not to be expected. If acute pancreatitis in laboratory animals follows a similar course, and there are indications that this is the case [26], then the focus should be on an effect on late mortality. A closer look at 3 out of 4 experiments reporting also a late mortality reveals that only the study of Van Minnen et al. [26] detected a significant reduction, whereas the others did not find any differences in late mortality. In other words, also on closer inspection, no overall positive or negative effects of probiotic supplementation on late mortality were found.

In light of the results presented above – no indications for harmful effects and quite strong evidence for efficacy – and given the regulatory requirements for food supplements (to which probiotics belong), it was defensible and understandable that the PROPATRIA-trial was started. However, there are some methodological issues which may hamper the interpretation of the experimental animal data and subsequently the translation to the clinical setting.

First of all, there were substantial differences in the design of the trial and the design of the animal experiments: a) all animal studies conducted before the start of the trial used other probiotic products than the one used in the trial, and in most cases single strain probiotics were tested in animals whereas PROPATRIA used multistrain probiotics Ecologic 641. After the start of the trial, some animal studies using Ecologic 641 have been published. However, none of these studies used both administration of probiotics after induction of AP and outcome measures comparable to the ones used in the PROPATRIA-trial. b) in the trial probiotics were administered in the jejunum whereas in most animal studies it was administered in the stomach, c) in 50% of the animal studies probiotic supplementation started before inducing AP, whereas in the PROPATRIA trial probiotica was supplemented to patients already suffering from severe AP. The exact physiological and translational significance of these differences is currently unclear and needs further study. Nevertheless, it is remarkable that an animal study with a study design similar to the human trial has not yet been conducted (i.e., an animal experiment studying the effect of Ecologic 641 supplemented in the jejunum after induction of AP on mortality and translocation/infection). In case such an experiment had been performed and the result had demonstrated an increased risk of mortality, we believe it would not have been responsible to start a clinical trial before conducting more similar experiments with a comparable design. In case this hypothetical experiment had shown no change or a diminished risk on mortality, a decision to go ahead would have been justified, also because a clinical trial with positive results of the use of probiotics in humans had been published already.

Second, the heterogeneity among the various animal studies is quite considerable. We tried to explain this heterogeneity through subgroup analyses (supplementation of probiotics before or after induction of AP, use of probiotics containing a single or multiple strains of bacteria), but in most cases these subgroup analyses did not substantially reduce the heterogeneity. Despite these limitations, the combined analysis still produced extra and useful information that could not directly be derived from the individual studies. Subgroup analyses showed for example that single strain probiotic supplementation might be more effective in reducing the overall histopathological score of the pancreas and the risk of bacterial translocation, as compared to multistrain supplementation. In future animal studies and clinical trials it seems worthwhile to investigate the possible difference in efficacy between mulitstrain and single strain probiotic mixtures.

Third, at the time that the PROPATRIA trial started only a few experimental animal studies had been published (n = 4) with relatively few animals per study. As a consequence, the power of the meta analysis, and thereby the reliability of the conclusions (the overall effect sizes), is relatively low. This limitation applies even more strongly to the subgroup analyses. For that reason, particularly the conclusions of the subgroup analyses should be treated as indications that elicit further investigation, rather than as hard conclusions.

Fourth, poor reporting of crucial pieces of information in the original manuscripts is of serious concern, and is also addressed by others [7], [8], [9], [28], [29]. This seriously hampers drawing reliable conclusions from animal studies. The risk of bias assessment and the table describing the characteristics of the included studies have clearly shown that crucial pieces of information (such as the timing of probiotic supplementation relative to the induction of AP, the number of animals per group included in the statistical analyses and the reasons for excluding animals) are often not well reported. Furthermore, only half of the studies reported that they blinded the outcome assessment and only one study provided sufficient details to judge the adequacy of the method of randomization. If the other studies did not actually blind the outcome assessment and if the methods of randomization used in the other studies were inadequate, there is a substantial risk that the effects of probiotics have been overestimated (see also [11]). If the actual effects of probiotics are indeed smaller than reported, it remains to be seen whether these effects are still statistically significant.

All in all, this SR has demonstrated that combining the results of all relevant animal studies published before and after the trial does not show an adverse effect of probiotic supplementation on the main outcome measure mortality, and shows a positive effect on the other outcome measures (reduced histopathology of the pancreas and BT to the pancreas and MLNs). In addition, subgroup analyses revealed that single strain probiotic supplementation might be more effective in reducing the overall histopathological score of the pancreas and the risk of bacterial translocation, as compared to multistrain supplementation. Therefore, we conclude that a more thorough analysis of all relevant animal studies (published both before and after the study protocol of the PROPATRIA trial) could not have predicted the harmful effects of probiotics supplementation as found in the PROPATRIA-trial.

However, it has also become clear that no animal study followed the experimental design of the human PROPATRIA trial as closely as possible, i.e., studying the effect on mortality and infection of supplementation in the jejunum of Ecologic 641 after induction of AP, which compromises a fair comparison between the results of the animal studies and the PROPATRIA trial. Moreover, a substantial portion of the animal studies had methodological shortcomings: insufficient power, potentially inadequate methods of randomisation and absence of blinding. In other words, the animal experiments were not executed and evaluated in such a way that the chances of a successful translation to the clinic were maximal. Yet, it is vital, both to justify the suffering of laboratory animals and to prevent harm in human patients, that the animal experiments conducted prior to clinical application are carried out in such a way that they yield the most and most reliable information. To prevent misunderstanding, there might be other factors that may explain (part of) the extrapolation problems. For example, there might be immunological differences between humans and other animal species as well as differences in the type of pancreatitis. These differences may further interfere with extrapolation of outcome data from animals to humans. It is too early, however, to conclude that animal experiments either can or cannot reliably inform regarding the potential outcome of human studies. Before drawing such a conclusion, it is important to first optimize the execution of preclinical animal experiments (through a design as close as possible to the intended clinical application, clear and clinically relevant primary outcome measures, proper power calculation, etc.). Accordingly, we strongly recommend a broad and explicit debate about the standards of animal experiments preceding clinical application.

## Supporting Information

Table S1 **Risk of bias of individual studies.** yes = low risk of bias, no = high risk of bias, ? = unclear risk of bias, n.a. = not applicable. * = assesment of the oucome measure histopathology was blinded, other relevant outcome measures were not blinded. ^∧∧^ Risk of bias in the analysis because animals were replaced. # solely animals with severe AP are included in the analysis (risk of underestimating the effect of probiotics).
(XLS)
